# Coping strategies as moderating factors to compassion fatigue among critical care nurses

**DOI:** 10.1002/brb3.1264

**Published:** 2019-03-18

**Authors:** Marwa A. Al Barmawi, Maha Subih, Omar Salameh, Najah Sayyah Yousef Sayyah, Noordeen Shoqirat, Raid Abdel‐Azeez Eid Abu Jebbeh

**Affiliations:** ^1^ Faculty of Nursing, Nursing Department Alzaytoonah University of Jordan (ZUJ) Amman Jordan; ^2^ Nursing Faculty Mutah University Karak Jordan

**Keywords:** burnout, compassion fatigue, coping strategies, critical care nursing

## Abstract

**Purpose:**

This study measured levels of compassion fatigue, burnout and satisfaction among critical care and emergency nurses. It investigated coping strategies as moderating factors and as predictors to levels of compassion fatigue.

**Methods:**

Using a cross–sectional design, this study was conducted on 228 (84.4%) out of 270 from four Jordanian hospitals. Nurses worked in different types of critical care units and emergency departments. Nurses completed a demographic questionnaire on the professional quality of life and coping strategies indicator scales.

**Results:**

Nurses had low to average compassion satisfaction, burnout and secondary stress syndrome. Problem‐solving and avoidance ranged between very low and average levels. Nurses reported having very low to average levels on seeking social support scale. Female nurses had better compassion satisfaction compared with their male colleagues, and the type of unit had a significant impact on the secondary stress syndrome, problem‐solving, and seeking social support. Nurses from the surgical cardiovascular ICU scored the highest mean scores on the secondary stress syndrome. Better coping strategies were associated with higher compassion satisfaction and lower levels of secondary stress syndrome. Problem‐solving significantly predicted compassion satisfaction, avoidance significantly predicted secondary traumatic syndrome.

**Conclusions:**

Coping strategies are moderating factors that could improve compassion satisfaction among critical care nurses. Managers could use findings to create healthier and supportive work environments. We recommend focusing on activities that promote better coping strategies, including improving the social support system. We also recommend replicating this study using a qualitative approach to identify further causes of compassion fatigue.

## 1 INTRODUCTION

Nursing is a discipline involving distinguished services characterized by its human nature. Nurses connect uniquely and therapeutically to their patients and family members (Potter et al., 2010). However, the closer nurses get to their patients while caring for them, things become more when an unexpected event takes place, including permanent loss of an organ, or a patient's death (Duarte, Pinto‐Gouveia, & Cruz, 2016). Nurses have always had exposure to a range of tragedies experienced by patients and family members (Beck, 2011). Their reactions to others’ profound losses or sudden unexpected deaths, especially in the critical care and emergency departments, have not, yet, received much consideration (Boyle, 2011; Kelly & Lefton, 2017). Compassion fatigue (CF) might develop because of the exposure to these traumatic events (Cieslak et al., 2014).

Figley (1995, 2012) suggested that frequent exposure to loss gives the carer the sense of CF, which could be described as “the cost of care”. Compassion fatigue, conceptually, refers to a state of emotional exhaustion, which can result from the intimate relationship with an individual or a group of individuals who had suffered a sudden or severe loss (Showalter, 2010).According to Adams, Figley, and Boscarino (2008), CF is a natural feeling and behavior arising from knowing the traumatizing events, which a significant other (e.g., a patient) has experienced. The level of stress then arises from helping or wanting to help that traumatized person, and this level increases up to a higher rate especially when help is unavailable or limited or the individual dies or is badly affected (Hiçdurmaz & Inci, 2015).

As nurses develop CF, it is expected that trajectories of this unbalanced sense of being would inevitably influence their lives, their decisions and inappropriate responses and might lead to more sick leave thus risking patient safety, with quality of care lessening and job dissatisfaction increasing (Fernandez‐Parsons, Rodriguez, & Goyal, 2013). It is necessary for nurses to enjoy emotional stability, mental alertness and physical wellbeing when practising their nursing. Therefore, investigating nurses’ well‐being is important to stimulate productivity, retention of posts within the discipline and more importantly to promote the provision of safe, quality care (Bellagamba, Gionta, Senergue, Bèque, & Cher‐Michel, 2015).

The recent changes in how patients perceive the quality of their nursing care, coinciding with the impact of changes in nursing practice during the past two decades have become additional sources of stress to nurses (Bellagamba et al., 2015). The speed of change in nursing practice and the aims of health care have a considerable impact on nurses’ reactions, which usually appear in the form of decreased ability to make sound clinical decisions and lessening of interest in the profession leading to intentions to leave the profession (Shang et al., 2014).

The frequent exposure to dramatic events and unexpected death could precipitate a sense of hardship, physical and mental exhaustion, and the need for a period of dissociation from the surroundings among critical care nurses (Van Mol, Kompanje, Benoit, Bakker, & Nijkamp, 2015). These exposures could lead to burnout, which refers to the occupation–related stress occurring among nurses results in demanding and emotionally charged relationships between those professionals and the care recipients, which may lead to personal, social, professional and psychological problems (Maslach & Jackson, 1986). All of which are symptoms that could be related to feelings of CF. This is especially true among nurses, who are devoted to their work and patients, and generally have positive attitudes toward their profession (Breslau & Schultz, 2013).

In part, compassion fatigue could explain changes in nurses’ sense of high frustration and job dissatisfaction, which may lead to lateral violence and an overall unhealthy work environment (Fernandez‐Parsons et al., 2013). There is a need for research investigating and suggesting interventions that tackle the ramifications of exposures that are repetitively present for health care workers, critical care nurses in particular (Kelly & Lefton, 2017). However, there is a need to investigate the presence of these symptoms as soon as they occur.

Studies exploring the impact of social and economic changes in Jordan on nursing care in general, and specifically compassion, are rare. Compassion fatigue, which describes the vicarious frequent exposure to trauma, has not yet been examined within the Jordanian context. In the light of this, devising strategies aiming at maximizing nurses' compassion satisfaction is a hard objective to achieve. This study explores this CF and coping strategies among Jordanian critical care nurses.

## 2 METHODS

### 2.1 Design

A cross–sectional design was used in this study to measure compassion fatigue and coping strategies among nurses working in critical care units.

### 2.2 Participants and settings

Data were collected from four governmental hospitals rated to be teaching hospitals. Nurses were working in twelve different units within these hospitals. Candidates eligible to participate in this study were nurses working in critical care units and emergency departments. Participants had to have at least two years of experience working in one of the critical care units. A total number of 270 questionnaires were distributed among the candidates, and 228 (84.4%) nurses returned the completed study questionnaire.

### 2.3 Ethical considerations

Ethical approval was obtained from the Ministry of Health (ref. no. 11/313/2016). Nurses chose to participate voluntarily and had the right to withdraw anytime. The data collected did not contain any names or descriptions that could identify any of the participants. The results were treated collectively as no indicators were used that could identify the unit or the hospital.

### 2.4 Procedure

After obtaining the approvals necessary to collect data from the Ministry of Health, one of the researchers approached unit managers and explained the study procedure and purposes. Managers granted permission to collect data, and the researchers talked to nurses in their units. Eligible nurses, who wanted to participate, were asked to take a copy of the study questionnaire; they were instructed to finish it within three weeks and return it into a box made for this purpose by the manager's office. The researchers collected the data themselves; no data collectors were hired in this study. Data were collected between April and December 2017.

### 2.5 Measures

Firstly, all necessary approvals to use the research instruments adopted in this study were obtained from the relevant authors, and all reserved rights and ethical issues concerning this use was followed. The study questionnaire was composed of three parts. The first part consisted of nonidentifying questions about the characteristics of the participants that could have influenced the findings and that could distinguish compassion fatigue (CF), levels of burnout (BO) and the coping strategies among nurses. The second part was the Professional Quality of Life Scale (ProQoL‐5) that measures compassion fatigue and satisfaction simultaneously.

The last part consisted of the Coping Strategies Indicator Questionnaire (CSI), which questions that determine the strategies adopted by nurses to cope with CF. Below is a more detailed explanation of the instruments adopted in this study. All instruments have been used with the permission of the authors, who will receive a copy of the results if requested, after finishing the study.

#### 2.5.1 The Professional Quality of Life Scale

The Professional Quality of Life Scale version 5 (ProQoL‐5) has 30 items and represents attempts to combine earlier subscales on compassion satisfaction with compassion fatigue (Stamm, 2012). It has three subscales; namely, the compassion satisfaction, a section that evaluates the pleasure clinicians derive from their work secondary to being exposed to traumatizing situations; the compassion fatigue or secondary traumatic stress items evaluate potential distress due to exposure to a range of different traumatized clients (i.e., critical care patients); and the burnout items evaluate feelings of hopelessness and frustration after little accomplishments (Sacco, Ciurzynski, Harvey, & Ingersoll, 2015).The range for the ProQOL‐5 subscales’ scores is 10–50.The subscales have been reported to have statistically acceptable internal consistency values, ranging from 0.75 to 0.88 (Stamm, 2002). The ProQol‐5 asked trauma clinicians (i.e., critical care nurses) to answer all items; the higher scores indicate a more negative psychological impact. Furthermore, the ProQOL‐5 measures both positive and negative impacts, and how both facets are related. Compassion satisfaction is composed of ten items. Compassion fatigue is divided into two subscales of ten items each: burnout is regarded as feelings of hopelessness and difficulty dealing with work or doing a job effectively, and secondary traumatic stress is regarded as a fright response resulting from extreme exposures by caregivers to traumatic events. Items are evaluated on a five‐point Likert's scale (from 1 = Never to 5 = Very Often) (Sansbury, Graves, & Scott, 2015).

According to Stamm (2012), the scores above 17 on the compassion fatigue subscale or a 27 on the burnout subscale show that the respondent is at higher risk for severe traumatic response. This instrument has been frequently used in many nursing studies (Kim, 2013; Smart et al., 2014).

#### 2.5.2 The coping strategy indicator

The coping strategy indicator (CSI) questionnaire has been developed and validated empirically through frequent use in many countries and over many years (Amirkhan, 1990). The CSI is composed of 33 items, with three scales of 11 items each: problem‐solving; seeking social support; and finally, avoidance (Amirkhan, 1994). These scales tap into the coping strategies common to a wide diversity of people dealing with a broad range of problems (Amirkhan& Auyeung, 2007).Responses on the scales range between 1 (not at all), 2 (a little), and 3 (a lot). The score for the CSI is between 33 and 99, and between 11 and 33 for the three scales.

## 3 DATA ANALYSIS

Data were analyzed using SPSS, version 21 (SPSS@IBM). The characterisitcs of the participants and scores on the study scales were identified using descritpive statistics. As normality of scores was established using normality tests, *t* test, ANOVA, and Pearson’s correlation were used to determine the link between the study variables, and the impact of participants’ characteristics on the study scales. The study also tested predictability between compassion fatigue and coping strategies using regression.

## 4 RESULTS

### 4.1 Characteristics of the participants

The age of the participants averaged between 21 and 51 with a mean age of 29.4 years. There were 123 (53.9%) females and 105 (46.1%) male participants. A majority of nurses had the baccalaureate degree as their highest nursing qualification. Nurses worked in different critical care units, including the Emergency Department, surgical ICU, and burn ICU (Table 1). Additionally, all nurses indicated that they had not participated in stress managing activities during the past two years.

**Table 1:** Characteristics of participants in the study (*N* = 228)

Factor	Categories	*N*	%
Sex	Male	123	53.9
	Female	105	46.1
Age (Mean 29.40, *SD* 5.684)	21–30	141	61.8
	31–40	80	35.1
	41–51	7	3.1
Nursing degree	Diploma	38	16.7
	Bachelor	177	77.6
	Masters	13	5.7
Unit	Emergency Department	77	33.8
	Medical ICU	35	15.4
	Surgical ICU	50	21.9
	CCU	31	13.6
	Burn ICU	35	15.4
Type of shift (hr)	8	181	79.4
	12	47	20.6
Weekly work hours	35–44	84	36.8
	45–60	119	52.2
	>60	25	11

### 4.2 Findings from the PROQOL‐5 and CSI findings

Mean scores were normally distributed with acceptable internal consistency (Table 2). Nurses’ responses on the ProQoL‐5 scales were analyzed, and the results showed that most nurses had low to average compassion satisfaction. Nurses also reported having low to average levels of burnout and secondary stress syndrome on the ProQoL‐5.

**Table 2:** Participants’ findings on the ProQoL‐5 and the CSI scales

	Mean	*SD*	Category	*N*	%	α^a^
PROQOL‐5						0.76
Compassion satisfaction	37.04	7.35	Low	181	79.4	0.82
			Average	47	20.6	
Burnout	28.87	4.57	Low	227	99.6	0.68
			Average	1	0.4	
Secondary stress syndrome	32.18	8.89	Low	217	95.2	0.76
			Average	11	4.8	
Coping strategy indicator	57.03	10.07				0.86
Problem‐solving	17.81	4.48	Very low	97	42.5	0.82
			Low	88	38.6	
			Average	43	18.9	
Avoidance	17.68	3.60	Very low	45	19.7	0.76
			Low	108	47.4	
			Average	74	32.5	
			High	1	0.4	
Seeking social support	19.82	3.933	Very low	81	35.5	0.69
			Low	119	52.2	
			Average	28	12.3	

a Cronbach's alpha.

Similarly, the mean scores on the CSI scales represented normal distribution and had acceptable levels of internal consistency (Table 2). Nurses in this study reported having various categories of problem‐solving and avoidance ranging from very low (42.5% and 19.7%, respectively) to average (18.9% and 32.5%, respectively). As well, the scores for nurses on the seeking social support scale ranged between very low (35.5%) to average levels (12.3%).

### 4.3 The impact of nurses’ characteristics, ProQoL‐5 and CSI findings

There was a significant effect of the participants’ sex on the compassion satisfaction scale (*p *= 0.012). Female nurses reported better compassion satisfaction compared with their male colleagues (Table 3). In this study, there was no statistically significant impact of nurses’ sex and the CSI scales (*p *> 0.05).

**Table 3:** The impact of nurses’ sex on the ProQoL‐5 and the CSI scales

**Scale**	Sex	Mean	*SD*	Sig.
Compassion satisfaction	Male	35.91	7.15	0.01
	Female	38.36	7.40	
Burnout	Male	29.07	4.41	0.48
	Female	28.64	4.76	
Secondary stress syndrome	Male	32.37	6.53	0.65
	Female	31.96	7.32	

The type of unit had a statistically significant impact on the compassion satisfaction (*p* = 030), as nurses working in medical ICU scored higher than nurses from other units. Type of unit had also statistically significant impact on the problem‐solving (*p *= 0.016), seeking social support scales (*p *= 0.010), and avoidance (*p *= 0.045) from the CSI. Nurses from the medical ICU scored the highest mean scores on the compassion satisfaction scale (Table 4). Nurses working in the surgical ICU and burn unit scored the highest on problem‐solving, surgical ICU nurses scored the highest on the seeking social support scale, and nurses from the burn ICU scored the highest on avoidance scale. However, there was no statistically significant relationship between ProQoL‐5 or CSI scales and the age of the nurse or the academic degree (*p* > 0.05).

**Table 4:** The impact of type of unit on the ProQoL‐5 and the CSI scales

	Compassion satisfaction		Burnout		Secondary stress syndrome	
	Mean	*SD*	Mean	*SD*	Mean	*SD*
ED	36.94	7.20	29.75	4.20	33.30	6.38
Medical ICU	**39.09**	7.72	28.00	4.43	33.51	7.29
Surgical ICU	35.53	5.23	29.65	3.69	32.37	5.38
CCU	38.81	6.12	27.55	5.27	29.90	8.41
Burn ICU	35.51	9.92	27.77	5.47	29.83	7.10
Sig.	0.030		0.361		0.993	

	Problem‐solving		Seeking social support		Avoidance	
	Mean	*SD*	Mean	*SD*	Mean	*SD*
ED	17.83	4.51	17.74	3.96	19.60	3.83
Medical ICU	17.17	3.55	17.06	2.96	19.43	3.83
Surgical ICU	**18.94**	3.96	**18.49**	2.99	19.51	3.26
CCU	15.48	3.16	16.16	3.58	19.61	3.58
Burn ICU	**18.94**	6.03	18.46	3.82	**21.46**	5.07
Sig.	0.016		0.010		0.045	

The bold items represent the highest mean scores.

As illustrated in Table 5, higher mean scores on problem‐solving, avoidance and seeking social support subscales on the CSI were associated with higher mean scores on the compassion satisfaction scale of the ProQoL‐5 using ANOVA (*p *< 0.001, *p *= 0.033, *p *= 0.011, respectively). These findings indicate that better coping strategies were associated with better levels of compassion satisfaction. In addition, higher mean scores on the avoidance and seeking social support subscales were associated with lower mean scores on the secondary stress syndrome (*p *= 0.001, *p *= 0.010, respectively). However, CSI scales did not have any statistically significant effect on the burnout scale on the PROQOL‐5 scales (*p *> 0.05).

**Table 5:** The impact of CSI scales on the compassion satisfaction and secondary stress syndrome scales

	Scale	Mean square	*F*	Sig.
Compassion satisfaction	Problem‐solving	8.56	16.30	0.000
	Avoidance	2.36	4.59	0.033
	Seeking social support	2.72	6.54	0.011
Secondary stress syndrome	Problem‐solving	1.85	3.33	0.070
	Avoidance	5.37	10.69	0.001
	Seeking social support	2.83	6.81	0.010

### 4.4 Predictors of compassion satisfaction and burnout among critical care nurses

Linear regression analysis was used to test if any of the CSI scales significantly predicted participants' compassion satisfaction. The results of the regression indicated the one predictor explained 27% of the variance (*R* ^2^ = 0.073, *F*(3,224) = 5.863, CI = 95%, *p* *= *0.001). It was found that problem‐solving significantly predicted compassion satisfaction (*β* = 0.123, *p *= 0.002), but not for avoidance and seeking social support scales (Table 6).

**Table 6:** Coping strategies as predictors to compassion satisfaction and secondary stress syndrome

	*R*	*R* ^2^	Unstandardized Coefficients		Sig.
			*b*	*SE b*	
Model 1	0.270	0.073			
(Constant)			1.517	0.093	0.000
Problem‐solving			0.123	0.040	0.002
Avoidance			−0.004	0.043	0.916
Seeking social support			−0.048	0.046	0.296
Model 2	0.229	0.052			
(Constant)			1.213	0.050	0.000
Problem‐solving			−0.004	0.021	0.855
Avoidance			−0.049	0.023	0.032
Seeking social support			−0.030	0.025	0.223

Note

Model 1: Compassion Satisfaction. Model 2: Secondary Stress Syndrome.

Predictors: Seeking Social Support, Problem‐solving, Avoidance.

Additionally, results of the regression indicated the one predictor explained 22.9% of the variance (*R* ^2^ = 0.052, *F*(3,224) = 4.134, CI = 95%, *p *= 0.007). It was found that avoidance significantly predicted secondary traumatic syndrome (*β* = −0.049, *p *= 0.032), but not problem‐solving or seeking social support scales. On the other hand, coping strategies could not predict burnout when measured by the burnout scale on the PROQOL‐5 among critical care nurses in this study (*p *> 0.05).

## 5 DISCUSSION

This study contributes to the pool of known knowledge by exploring the impact of coping strategies on CF in a sample of Jordanian critical care nurses. This study examined the prevalence of CF using ProQol‐5 and coping strategies using CSI. The current study extends the literature documenting the link between CS, BO and SSS. It further investigated the link between coping strategies and the compassion fatigue among in ten different critical care units in Jordan. Finally, it used findings from both scales to examine the level of predictability of coping strategies as moderating factors CF.

The CF found in this study was at a lower level than the literature reported (Karanikola et al., 2015). The average CS, BO and SSS scores categorize nurses’ responses in this study as being low to average levels. In the literature, various studies findings reported that most nurses in critical care units had moderate to high levels of burnout and CF (Hooper, Craig, Janvrin, Wetsel, & Reimels, 2010; Kelly & Lefton, 2017). Similarly, another study by Hunsaker, Chen, Maughan, and Heaston (2015) found that emergency nurses experienced low to average levels of BO and CF but had an average to high level of satisfaction.

Elkonin and Van der Vyver (2011) suggest that ICU nurses are at a high risk for compassion fatigue, a moderate risk for BO and low level of CS. They also reported a negative relationship between CS and BO or CF, but the relationship between CF and BO was positive which supports the findings in this study (Elkonin & Van der Vyver, 2011). There is the possibility that the elevated levels of CF and BO influence nurses’ productivity and care making a negatively (Fernandez‐Parsons et al., 2013). Lee, Lee, Gillen, and Krause (2014) found that critical care nurses usually complained of physical symptoms, such as exhaustion and musculoskeletal pain, secondary to their experiencing compassion fatigue and burnout due to high stress levels. These symptoms often result in high rates of absenteeism as well as presenteeism, which means that nurses are there in body but not aware of the surrounding (Umann, Guido, & Grazziano, 2012).

In this study, female nurses reported experiencing better CS compared with their male colleagues. Opposite to findings in this study, Robins, Melizer, and Zelikovsky (2009) found that although the coping strategies were linked to CS there were no other no significant gender differences in SSS. Another study found that nurses’ sex did not influence levels of BO, or CS (Hooper et al., 2010). One exception, however, was that female nurses expressed higher levels of CF than the males. In Taiwan, Tzeng, Chung, and Yang (2013) found that the younger nurses reported better satisfaction with work. There are contradicting findings in the literature, which might be related to the interference of other variables, which were not included in these studies, such as relationships with colleagues and managers, financial and marital status, the presence of children, and so on.

In a USA study, Dominguez‐Gomez and Rutledge (2009) reported that many emergency nurses experienced irritability, avoided patients, and had intrusive thoughts about patients as related to a sense of BO. Although the majority of nurses participated regularly in stress management and self‐care activities, many reported having at least one stress related symptom during the last 7 days despite participating in stress management, demonstrating they had experienced CF. In our study, no nurses reported participating in stress managing activities, in fact only 33% of nurses met the criteria of SSS. In our study, the highest levels of SSS were found among nurses working in the surgical ICU. The main issue is that nurses’ clinical reasoning and judgment could be affected by mental stress leading to inaccurate clinical decisions and a reduction in nurses’ practical delivery of care (Kim, Han, Kwak, & Kim, 2015).

Higher mean scores on problem‐solving, avoidance and seeking social support subscales on the CSI were associated with higher levels of CF, BO and SSS. In other words, better coping strategies were associated with better levels of CS. Supporting these findings, Chang (2012) suggested that emotion‐focused coping fully mediated the relationship between maladaptive perfectionism and burnout. Another study from South Korea found that emotional exhaustion and depersonalization among nurses was reduced when emotion–focused and problem–focused coping strategies were used (Shin et al., 2014). A broad analysis of the findings in this study emphasized what many authors, like Jenkins and Warren (2012) and Kelly, Runge, and Spencer (2015), who remarked that nurses’ feeling of CF can lead to poor judgment, loss of empathy, and decreased productivity, risking patient safety and lowering quality of care.

Higher mean scores on the avoidance and seeking social support subscales were associated with lower mean scores on the secondary stress syndrome. This finding indicated the importance of a social support system within the work environment, which is another factor influencing nurses’ ability to accommodate the stressful nature of the critical care setting. The literature also suggested that a reduction in the level of avoidance leads to lower stress levels (Neff & Germer, 2013). Findings in the present study may be extrapolated to suggest that difficulties with coping strategies are the key explanatory mechanism underlying the presence of BO and CF. Another report in the literature suggested that higher levels of BO and dissatisfaction were associated with a poor work environment and the social support found to be lacking (McHugh, Kutney‐Lee, Cimiotti, Sloane, & Aiken, 2011). This environment with a weak social support system could promote higher levels of CF among nurses (Thompson, 2013).

Problem‐solving significantly predicted CS, and avoidance significantly predicted SSS. Better levels of satisfaction were achieved by improved problem–solving strategies, which aided the nurses to address the sources of stress (Potter, Deshields, & Rodriguez, 2013). While this study focused mainly on exploring coping strategies in nurses, other studies addressed issues concerning the work environment and other personal characteristics of the nurses, such as age and years of experience, which some researchers reported as significant predictors of BO (Kelly et al., 2015).

Individuals from diverse cultures working in the same profession may experience BO differently, in terms of levels of emotional exhaustion, personal accomplishment and depersonalization as discussed by Cieslak et al. (2014). Therefore, the use of a scale that is culture–sensitive might achieve more representative findings about CF and BO among critical care nurses. Although widely used in research, the standardized cut offs for the ProQOL‐5 scores might not be reflecting the nature of nursing work or the nurse population, as the scale was used among a range of professions. We suggest that more research should be conducted to determine if nurses consistently average high or low scores on the scale.

## 6 CONCLUSIONS

This study documented compassion fatigue and coping strategies as moderating factors among critical care nurses in Jordan. Coping strategies were significant predictors and represented powerful indicators to the level of compassion satisfaction and correlated negatively with compassion fatigue. The presence of significant levels of stress and BO point to the need for future research to explore this area further. This is particularly important, as there are statistically significant relationships between CSI scales and the ProQOL‐5 scales; however, not all relationshipsare proven predictors. Therefore, some of these relationships are still not well defined, and require further investigations to define them.

Findings in this study cannot be conclusive and require that the other potential causes of CF and BO be examined putting in mind that the predictive nature of findings in this study, using coping strategies, is limited and more factors should be explored. Additionally, other coping strategies could be included in future studies including the use of organizational support, spirituality–related practices, the presence of counseling services at the hospital, and the use of exercise, and meditation.
